# Lifestyle and environmental risk factors associated with cancer: A case-control study in Bangladesh

**DOI:** 10.1371/journal.pone.0328745

**Published:** 2026-01-28

**Authors:** Mohammad Lutfor Rahman, K. M. Tanvir, Farzana Rahman, Sreshtha Chowdhury, Shuvajit Saha, Md Abdullah Saeed Khan, Mohammad Azmain Iktidar, Mehedi Hasan, Samiha Nahar Tuli, Sharmin Akter, Tajrin Rahman, Mohammad Hayatun Nabi, Mohammad Delwer Hossain Hawlader

**Affiliations:** 1 Institute of Statistical Research and Training, University of Dhaka, Dhaka, Bangladesh; 2 Department of Epidemiology and Community Health, University of North Carolina at Charlotte, Charlotte, North Carolina, United States of America; 3 Department of Epidemiology, Florida International University, Miami, Florida, United States of America; 4 Usher Institute, University of Edinburgh, Edinburgh, United Kingdom; 5 National Institute of Preventive and Social Medicine, Dhaka, Bangladesh; 6 Directorate General of Health Services, Dhaka, Bangladesh; 7 Public Health Promotion and Development Society (PPDS), Dhaka, Bangladesh; 8 Department of Neurology, Dhaka Medical College and Hospital, Dhaka, Bangladesh; 9 Department of Public Health, North South University, Dhaka, Bangladesh; 10 NSU Global Health Institute (NGHI), North South University, Dhaka, Bangladesh; Shuguang Hospital, CHINA

## Abstract

Cancer remains the second leading cause of death worldwide, with cases rising at an alarming rate. While the causes of cancer are complex and varied, certain risk factors - such as exposure to environmental pollutants and specific lifestyle choices - are modifiable and can be addressed. A case-control study was conducted in Bangladesh from 25 August 2023 to 18 April 2024 to examine the association between cancer risk and a range of lifestyle and environmental factors. The study specifically focused on six common cancer types: breast, hematological, oral, cervical, colorectal, and lung cancer. This study identified several lifestyle and environmental factors positively associated with cancer risk. Individuals using wood or kerosene for cooking had higher odds of cancer compared to those using supplied gas (AOR = 3.886). Consumption of overcooked or poorly cooked food was associated with an increased risk of cancer compared to the consumption of well-cooked food (AOR = 2.478). Oral hygiene also showed a relationship, with participants brushing their teeth only 2-3 times a week having a higher chance of cancer compared to those who brush regularly (AOR = 3.103). In addition, frequent exposure to mosquito repellent was positively associated with cancer risk (AOR = 1.569), and exposure to inorganic dust showed a similar association (AOR = 1.673). These findings highlight modifiable lifestyle and environmental factors that could inform future cancer prevention strategies in Bangladesh.

## Introduction

Cancer is one of the potential causes of death worldwide, presenting a serious public health concern. The World Health Organization (WHO) reported that in 2018, Bangladesh experienced 150,781 new cases of cancer and 108,137 cancer-related deaths [1]. These alarming statistics highlight the pressing need for effective strategies to combat cancer, especially as projections suggest that both incidence and mortality rates are expected to increase in the coming decades. However, ongoing research underscores the potential for preventing a significant proportion of cancer cases through modifications in lifestyle and environmental risk factors [2].

A growing body of evidence shows that lifestyle behaviors play an important role in cancer prevention. Smoking cessation remains one of the most important preventive measures, as smoking is a well-established risk factor for multiple cancers, most notably lung cancer [3]. Maintaining a healthy body weight is crucial, as obesity has been associated with increased risk of several cancers. Regular physical activity significantly reduces the risk of cancers such as breast cancer among women [4]. A balanced diet rich in fruits, vegetables, and whole grains, and avoidance of processed foods, has also been linked to cancer prevention [5]. In addition, poor oral hygiene has been identified as a risk factor for oral cancers [6].

Beyond lifestyle factors, reducing exposure to environmental pollutants is also critical. Both indoor and outdoor air pollution may have been associated with an increased risk of cancer [7]. Pollutants such as particulate matter and industrial chemicals can contribute to cancer development [8,9]. Occupational hazards, such as exposure to asbestos, inorganic dust, and silica, have consistently shown positive associations with cancer risk [10]. Furthermore, pesticide exposure, especially in agricultural communities, has been recognized as an important contributor to cancer [11]. Other environmental factors, such as soil contamination, and temperature variations may have also been positively correlated with increased cancer risk [12].

Despite these insights, there remains a research gap concerning the comprehensive effects of lifestyle factors and environmental exposures on cancer prevention and prognosis, particularly in Bangladesh. Specifically, a significant proportion of rural households in Bangladesh rely on traditional biomass fuels such as wood, cow dung, and kerosene for cooking. According to the Bangladesh Demographic and Health Survey report (2017-18) [13], over 80% of rural households continue to use biomass fuels, exposing women and children disproportionately to hazardous smoke in unventilated indoor spaces [14]. This level of reliance is significantly higher than in many neighboring South Asian countries, where access to cleaner fuels has improved more rapidly [15].

These fuels release harmful particulate matter and carcinogenic compounds into indoor environments, leading to prolonged exposure to toxic fumes. The concentration of indoor PM_2.5_ from biomass fuel in these households has been measured at levels exceeding 1000 $\mu g/m^{3}$, far above the WHO recommended limit of 25 $\mu g/m^{3}$, indicating an extreme level of exposure [14,16]. Given that approximately 59% of the Bangladeshi population resides in rural areas where the use of biomass fuels is widespread [17], Bangladesh provides a particularly relevant setting for evaluating the long-term health impacts of indoor air pollution on cancer development, especially among women and elderly individuals who spend extended periods indoors near cooking stoves [18].

In addition, Bengali cuisine frequently involves the consumption of fish and meat prepared at high temperatures or over open flames. Improper cooking practices, particularly charring or overcooking, can generate carcinogens such as heterocyclic amines and polycyclic aromatic hydrocarbons, both of which have been linked to cancer [19–21]. The cultural practice of cuisine distinguishes the Bangladeshi population from other ethnic groups, providing a critical area for investigation in cancer research.

Bangladesh’s rapid industrialization has led to increased exposure to various environmental pollutants such as inorganic dust, silica, and pesticides. Many rural Bangladeshis are exposed to these harmful environmental substances due to industrial and agricultural activities and poor occupational safety standards [22]. In addition, the use of mosquito repellents is widespread, especially during the monsoon season, and these repellents have been linked to increased cancer risk due to their chemical components [23]. The impact of these environmental exposures on cancer biology in the Bangladeshi population may differ from other regions due to both the intensity and duration of exposure.

Furthermore, there is a lack of case-control studies that explore how adherence to specific lifestyle patterns and exposure to environmental factors influence cancer risk. To address these research gaps, this study aims to update and synthesize existing knowledge on these associations. By focusing on epidemiological studies conducted in Bangladesh, we seek to provide a clearer understanding of how lifestyle and environmental factors contribute to cancer risk, ultimately informing more effective prevention and intervention strategies.

## Materials and methods

### Data

A case–control study was carried out to examine the influence of dietary and environmental factors on cancer over an eight-month period (25 August 2023 to 18 April 2024). The study included patients diagnosed with one of six cancer types—breast, hematological, oral, cervical, colorectal, or lung—who were classified as cases. Controls were carefully selected to match cases on gender, age, and family background. To ensure age compatibility, each control’s year of birth was required to fall within ±10 years of the corresponding case. Whenever feasible, controls were chosen from the same family to enhance genetic comparability. All controls were confirmed to be free of cancer and major comorbidities through screening based on medical history and self-reported absence of any past or present cancer diagnosis.

A structured questionnaire was developed to capture relevant variables, including demographic factors (age, gender, educational qualification, residence, marital status, and religion), lifestyle characteristics, environmental exposures, and dietary habits. This questionnaire was rigorously reviewed and refined by experts and was updated following a pilot survey conducted at National Institute of Cancer Research and Hospital (NICRH).

The sample size for a 1:1 matched case–control study was estimated using the Dupont (1988) method [24]. Assuming a two-sided significance level of 0.05, 80% power, and an expected exposure prevalence of 20% among controls, the required number of pairs to detect a matched odds ratio of 2.0 was calculated to be approximately 350 pairs. Data collection was carried out across the country at 20 reputable cancer hospitals, selected based on their high patient volume, availability of oncology services, and geographical accessibility. Within each hospital, proportional sampling was applied to capture variability in patient demographics. Intern doctors, who were thoroughly trained prior to the study, were assigned to collect the data.

We successfully collected a total of 713 responses, with 361 from the case group. For each case, one control was intended to be matched; however, due to challenges in finding appropriate controls, nine cases did not have matched controls. To ensure data quality, completed questionnaires were checked on a regular basis for key variables by the field supervisor. In addition, a random subset of responses was re-validated through follow-up checks with participants to confirm accuracy and consistency. The collected data were then coded by a specialized data entry team.

### Variables

Dependent Variable: The dependent variable was binary, indicating whether a patient was diagnosed with one of six specific types of cancer: breast, hematologic, oral, cervical, colorectal, or lung cancer. The presence of any type of cancer was considered a case and coded as 1, while the absence of cancer (controls) was coded as 0.

Independent Variables: There were two categories of independent variables- lifestyle and environmental factors. Lifestyle variables included type of transportation used, leisure time activity, type of cooking fuel, method of cooking meat, frequency of teeth cleaning, drinking water habit before breakfast, exposure to smoke during cooking, type of food consumed, amount of oil in food and, frequency of cleaning or vacuuming at home. Environmental variables included exposure to engine exhaust, inorganic dust, cotton dust, silica, smog, pesticides, and mosquito repellent.

To ensure clarity and reproducibility, operational definitions were established for dietary and environmental exposure categories used in this study. Foods were classified as very oily if prepared with excessive amounts of oil, such as deep-fried items. Regular oily foods referred to dishes prepared with moderate amounts of oil typical of daily cooking, while less oily foods were those prepared with minimal or no added oil, such as boiled, steamed, or lightly sauteed items. Food items were categorized as regular spicy when prepared with standard levels of spice common in Bangladeshi diets, very spicy when prepared with an excessive amount of chili or strong spices. Cooking practices were defined as overcooked when items were visibly charred or cooked at high temperatures for prolonged periods, and inadequately cooked when items were partially cooked with uncooked portions still visible.

Environmental exposure variables were also standardized. Frequent exposure indicated daily or near-daily contact with a pollutant such as engine exhaust, inorganic dust, or silica, whereas rare exposure referred to occasional or infrequent contact, defined as once per week or less. These definitions were explained to participants during data collection to ensure consistency in responses. To minimize the potential recall bias in questionnaire responses, participants were provided with examples of key variables. Interviewers were trained to use standardized, neutral questioning techniques and structured prompts to reduce misreporting.

### Ethical considerations

This study adhered to the ethical guidelines of Dhaka Ahsania Mission’s Institutional Review Board (IRB)/Ethical Review Committee (ERC) (Approval No.: 2023/DAM/IRB/ERC/2302). The ethical standards outlined in the 1964 Helsinki Declaration and its later revisions were followed where applicable. Written informed consent was obtained from all participants during face-to-face interviews, ensuring they understood their participation was voluntary and could be withdrawn at any time before signing.

### Statistical analysis

The chi-square test was applied to assess the associations between categorical variables and cancer events. Multiple logistic regression was used to calculate adjusted odds ratios to evaluate the effects of lifestyle and environmental factors on cancer, while controlling for age, gender, and smoking status. All statistical tests were conducted with a significance level set at 5%. Statistical analysis was performed using SPSS 25.

## Results

Table 1 presents the distribution of the background characteristics for cases and controls. Statistically significant differences were observed between cases and controls in terms of educational attainment, occupational status, and marital status (p<0.05). In particular, educational levels differed significantly between the two groups: the majority of controls had higher levels of education, while the cases were more evenly distributed between various educational backgrounds. Furthermore, the study population was predominantly Muslim, with this characteristic being similarly distributed between both cases and controls. Other demographic and socioeconomic factors were also well distributed in cases and controls, indicating that the groups were comparable in these attributes. This distribution ensures that any observed differences in cancer risk factors are less likely to be attributable to discrepancies in these baseline characteristics.

**Table 1:** Sociodemographic characteristics of cancer cases and matched controls in the study population.

Category	Labels	Case (%)	Control (%)	p-value
Education	No education	76 (21.4)	23 (6.6)	<0.001
	Primary	98 (27.6)	30 (8.6)	
	Secondary	96 (27.0)	75 (21.5)	
	Higher	85 (23.9)	221 (63.3)	
Occupation	Govt employee	12 (3.4)	16 (4.7)	<0.001
	Non-govt employee	35 (9.9)	47 (13.8)	
	Business	46 (13.0)	47 (13.8)	
	Student	18 (5.1)	87 (25.5)	
	Others	242 (68.6)	144 (42.2)	
Religion	Muslim	326 (91.8)	322 (92.8)	0.632
	Non-Muslim	29 (8.2)	25 (7.2)	
Residence	Urban	128 (35.7)	149 (42.3)	0.068
	Rural	231 (64.3)	203 (57.7)	
Marital Status	Married	31 (8.7)	136 (40.1)	<0.001
	Unmarried	317 (89.0)	200 (59.0)	
	Others	8 (2.2)	3 (0.9)	

Table 2 details the distribution of individual lifestyle characteristics among cases and controls. Several key indicators related to lifestyle were significantly associated with cancer risk, including the type of fuel used for cooking, methods of cooking meat, and exposure to smoke during cooking (p < 0.001).

**Table 2:** The lifestyle characteristics and their distribution among cases and controls.

Variable	Case(%)	Control(%)	p-value
**Transportation type**			
Public	315(94.0)	271(93.8)	0.893
Private	20(6.0)	18(6.2)	
**Leisure time activity**			
Active works:Walking/Playing	62(21.2)	49(21.0)	0.896
Inactive works: Watching TV/ Social Media	231(78.8)	184(79.0)	
**Fuel type in cooking**			
Wood or Kerosene	212(59.7)	137(44.6)	<0.001
Supplied Gas	143(40.3)	170(55.4)	
**Cooking type of meat**			
Well Cooked	252(70.8)	270(86.3)	<0.001
Over or Inadequately Cooked	104(29.2)	43(13.7)	
**Frequency of cleaning teeth**			
Everyday	206(81.7)	174(83.3)	0.672
2/3 times a week	46(18.3)	35(16.7)	
**Drinking water habit before breakfast**			
Yes	88(33.6)	107(39.3)	0.130
No	174(66.4)	161(60.1)	
**Smoke during cooking**			
Yes	255(72.6)	168(50.9)	<0.001
No	96(27.4)	162(49.1)	
**Consumed food type**			
Regular Spicy	177(68.6)	176(67.2)	0.727
Very Spicy	81(31.4)	86(32.8)	
**Amount of oil in consumed food**			
Regular	197(75.8)	195(72.5)	0.202
Very or Less Oily	63(24.2)	74(27.5)	
**Sweep or vacuum home**			
Daily	22(6.4)	25(7.6)	0.554
<7 times a week	320(93.6)	304(92.4)	

This bivariate findings reveal that a higher proportion of cases used wood or kerosene as cooking fuel (59.7%) compared to controls (44.6%). This suggests a notable association between the type of cooking fuel and cancer risk (p < 0.001). Additionally, 29.2% of cases consumed overcooked or inadequately cooked meat in contrast to 13.7% of controls (p < 0.001). Exposure to smoke during cooking was also found to be significantly associated with cancer risk. A higher percentage of cases were exposed to cooking smoke (72.6%) compared to controls (60.9%), highlighting a strong association between smoke exposure and cancer incidence. Other lifestyle factors considered in the study did not show significant associations with cancer risk in this bivariate analysis.

Table 3 presents the associations between various lifestyle factors and cancer risk after adjusting for demographic variables, revealing several significant relations. The type of cooking fuel used was notably associated with cancer risk; specifically, individuals using wood or kerosene for cooking had 3.886 times odds of developing cancer compared to those using supplied gas (95% CI = 1.578–9.572), suggesting that the use of less clean cooking fuels may be linked with as increased cancer risk. Additionally, oral hygiene practices were significantly related to cancer risk, with individuals who brushed their teeth 2–3 times a week facing three times higher odds of cancer compared to those who brushed daily (95% CI = 1.021–9.428), indicating the impact of consistent oral care on cancer risk. Dietary habits also played a role: those consuming over or inadequately cooked food had a 2.478 times odds of cancer compared to those with well cooked food consumption (95% CI = 1.022-6.008), highlighting the potential benefits of consuming well cooked food.

**Table 3:** Multivariate-adjusted odds ratios and confidence intervals of cancer prevalence associated with lifestyle characteristics.

Variable	Adjusted OR	p-value	95% CI
**Transportation type**			
Public (Ref)	1	–	–
Private	0.463	0.373	(0.085, 2.522)
**Leisure time activity**			
Inactive works (Ref)	1	–	–
Active works	0.263	0.195	(0.035, 1.983)
**Fuel type in cooking**			
Supplied Gas (Ref)	1	–	–
Wood or Kerosene	3.886	0.003	(1.578, 9.572)
**Cooking type of meat**			
Well Cooked (Ref)	1	–	–
Over or Inadequately Cooked	2.478	0.045	(1.022, 6.008)
**Frequency of cleaning teeth**			
Everyday (Ref)	1	–	–
2/3 times a week	3.103	0.046	(1.021, 9.428)
**Drinking water before breakfast**			
No (Ref)	1	–	–
Yes	0.665	0.394	(0.260, 1.700)
**Smoke during cooking**			
No (Ref)	1	–	–
Yes	1.807	0.206	(0.722, 4.521)
**Consumed food type**			
Regular Spicy (Ref)	1	–	–
Very Spicy	1.022	0.960	(0.429, 2.434)
**Amount of oil in consumed food**			
Regular (Ref)	1	–	–
Very or Less Oily	0.498	0.133	(0.201, 1.237)
**Sweep or vacuum home**			
<7 times a week (Ref)	1	–	–
Daily	0.888	0.880	(0.190, 4.151)

Table 4 details the distribution of components of environmental exposures among cases and controls. Exposure to inorganic dust was significantly associated with cancer (p-value = 0.003) where 69.6% were cases and 58.0% were controls. Silica exposure also showed a strong association with cancer; 76.9% of cases were frequently exposed to silica, in contrast to 62.9% of controls, with the association being highly significant (p-value < 0.001).

**Table 4:** Environmental exposures and their distribution among cases and controls.

Variable	Case(%)	Control(%)	p-value
**Engine exhaust**			
Rarely	155(49.4)	173(54.7)	0.264
Frequently	159(45.3)	143(50.6)	
**Inorganic dust**			
Rarely	94(30.4)	133(42.0)	0.003
Frequently	215(69.6)	184(58.0)	
**Cotton dust**			
Rarely	50(16.3)	66(22.1)	0.079
Frequently	256(83.7)	232(77.9)	
**Silica**			
Rarely	69(23.1)	112(37.1)	<0.001
Frequently	230(76.9)	190(62.9)	
**Smog**			
Rarely	61(19.9)	72(23.4)	0.327
Frequently	246(80.1)	236(76.6)	
**Pesticides**			
Rarely	50(16.3)	62(20.5)	0.351
Frequently	257(83.7)	240(79.5)	
**Mosquito repellent**			
Rarely	146(43.6)	206(65.4)	<0.001
Frequently	169(53.7)	109(34.6)	

Additionally, the use of mosquito repellent was significantly linked to cancer risk, with 53.7% of cases frequently exposed to mosquito repellent compared to 34.6% of controls (p-value < 0.001). Other environmental factors examined did not demonstrate significant associations with cancer at the 5% level of significance. These results highlight the substantial impact of inorganic dust, silica, and mosquito repellent exposure on cancer risk, emphasizing the need for targeted interventions in these areas.

Table 5 presents the adjusted odds ratios and confidence intervals for cancer prevalence associated with various environmental exposures taking into account the rarely exposed group as the reference. Our analysis indicates that exposure to mosquito repellent is marginally associated with an increased risk of cancer. Individuals who reported frequent exposure had 56.9% higher odds of developing cancer compared to those with rare exposure (95% CI: 0.965–2.549). Similarly, exposure to inorganic dust showed a marginal association with cancer prevalence, with 1.67 times higher odds (95% CI: 0.913–3.064).

**Table 5:** Multivariate-adjusted odds ratios and confidence intervals of cancer prevalence associated with environmental factors.

Category	Adjusted OR	p-value	95% CI
**Engine exhaust**			
Rarely (Ref)	1	–	–
Frequently	0.749	0.288	(0.440, 1.276)
**Inorganic dust**			
Rarely (Ref)	1	–	–
Frequently	1.673	0.096	(0.913, 3.064)
**Cotton dust**			
Rarely (Ref)	1	–	–
Frequently	0.944	0.852	(0.519, 1.720)
**Silica**			
Rarely (Ref)	1	–	–
Frequently	0.701	0.220	(0.397, 1.237)
**Smog**			
Rarely (Ref)	1	–	–
Frequently	1.277	0.420	(0.704, 2.316)
**Pesticides**			
Rarely (Ref)	1	–	–
Frequently	0.768	0.415	(0.408, 1.447)
**Mosquito repellent**			
Rarely (Ref)	1	–	–
Frequently	1.569	0.069	(0.965, 2.549)

## Discussion

The current study investigated the association between lifestyle and environmental factors among cancer patients through a case-control study in Bangladesh. The primary objective was to identify potentially modifiable lifestyle risk factors that could help mitigate cancer prevalence. This study demonstrates significant associations between lifestyle and environmental exposures and elevated cancer risk in the Bangladeshi population. These findings align with prior research while highlighting context-specific risks relevant to low-resource settings, providing valuable insights for public health interventions.

The present study found a significant association between the method of cooking meat and cancer risk. Specifically, consuming overcooked meat was linked to an increased risk of cancer. This finding aligns with the European Food Safety Authority’s 2015 risk assessment, which indicated that overcooked food could elevate cancer risk across all age groups due to the formation of carcinogenic compounds such as heterocyclic amines and polycyclic aromatic hydrocarbons [19,25]. These compounds are formed particularly during frying, grilling, and roasting, processes that involve direct high-heat exposure. While most of the existing evidence comes from Western populations, our study provides important insights from Bangladesh, where frying and open-flame cooking of fish and meat are widespread, potentially leading to even greater exposure to heterocyclic amines and polycyclic aromatic hydrocarbons (PAHs).

Similarly, the association observed between consumption of undercooked meat and cancer risk may be explained by microbial and parasitic contamination, as insufficient cooking can allow pathogens such as Helicobacter pylori, Salmonella, or liver flukes to survive. Chronic infections from these organisms may have been linked to cancer through mechanisms involving persistent inflammation and cellular damage [26]. This evidence underscores the need for public awareness about safe cooking practices to reduce cancer risk.

In addition, we observed a significant relationship between cancer risk and the type of fuel used for cooking. Specifically, using wood and kerosene was associated with higher cancer risk. This is consistent with a study conducted in Iran, which reported that burning biomass or kerosene, especially in poorly ventilated areas, increased the risk of digestive cancers [27]. The observed link between wood and kerosene cooking fuels and cancer risk supports existing evidence that biomass combustion releases carcinogenic pollutants such as PAHs, fine particulate matter (PM2.5), and volatile organic compounds [14–16,28]. Prolonged indoor exposure, particularly in poorly ventilated rural households, likely intensifies this risk [14,29]. The use of such fuels contributes to indoor air pollution, which has been identified as a significant cancer risk factor. The current study also highlighted the adverse effects of cooking smoke on cancer risk. This finding is supported by research from Taiwan, which demonstrated that exposure to cooking fumes is associated with an increased risk of lung cancer [7]. Effective kitchen ventilation and smoke control are essential in reducing the potential health risks associated with cooking fumes.

Although the association between very oily or less oily food and cancer risk was not statistically significant, the lower odds ratio observed is noteworthy. This counterintuitive trend may be explained by residual confounding, as the regular oily category represents the most common dietary practice in Bangladesh and likely represents more frequent and larger consumption of oil-based foods. While high-fat diets and repeated frying have been linked to carcinogenesis through mechanisms such as obesity, chronic inflammation, and oxidative stress [5,30], occasional deep-fried intake or low-oil cooking methods may not produce the same risks. Therefore, this finding should be interpreted with caution, and larger studies with more detailed dietary assessment are needed to clarify the relationship between oil use in cooking and cancer risk.

Furthermore, this study observed a marginally non-significant association between exposure to inorganic dust and cancer risk. This finding is consistent with results from a study in Montreal, which reported an increased cancer risk associated with inorganic dust exposure [31]. Although statistical significance was not reached in our study, the established carcinogenic nature of inorganic dust underscores the need for protective measures in occupational and environmental settings where such exposures are common.

Finally, a significant association was found between cancer risk and the use of mosquito repellents. This finding is in agreement with research conducted in Taiwan, which reported a correlation between exposure to mosquito repellents and cancer risk [32]. The potential carcinogenic effects of some chemicals in mosquito repellents warrant cautious use and further investigation.

The increased risk associated with mosquito repellent use may be linked to chronic exposure to chemicals such as N,N-diethyl-meta-toluamide and permethrin, which exhibit genotoxicity in experimental studies [33,34]. In addition, exposure to cooking fumes containing fine particulate matter and aldehydes can trigger oxidative stress and inflammation, biological processes that are well recognized as contributors to cancer development [14,16].

These combined inhalational, dietary, and dermal exposures can act synergistically with genetic and nutritional factors. The unique environmental context of Bangladesh including widespread biomass fuels, traditional cooking practices, and high chemical exposure underscores the need for localized epidemiological evidence to guide targeted cancer prevention efforts [14,28,29].

### Limitations and future directions

There are several limitations in this study. For instance, the reliance on self-reported data may have introduced recall bias, potentially affecting the accuracy of exposure classification. Although structured questionnaires and clear definitions were used to minimize this risk, some degree of misclassification remains possible. In addition, the use of family-based controls, while reducing genetic and socio-cultural variability between cases and controls, may have introduced selection bias and limits the generalizability of our findings to the broader population. Moreover, although controls were matched to cases by age and sex, other important confounders—such as occupation, income, and place of residence—were not matched. This limitation may have led to residual confounding, which should be considered when interpreting the results.

The limited sample size also restricted our ability to perform stratified analyses by cancer type. Although significant associations were observed between exposures such as cooking fuel or mosquito repellents and overall cancer risk, we were unable to determine whether these relationships varied across cancers with distinct etiologies. Furthermore, the relatively wide confidence intervals observed for some exposures, such as cooking fuel type, likely reflect the limited sample size and the small number of participants within certain categories. Larger studies with greater statistical power are therefore needed to obtain more precise effect estimates and to confirm these associations.

Although we matched cases and controls by age and sex and adjusted for smoking status in the multivariate model, we were unable to adjust for other potential confounders such as occupation and income. In addition, cumulative long-term exposure to lifestyle and environmental factors could not be measured. Besides, we were unable to capture the duration and intensity of exposures, which may be critical in understanding cancer risk. Complete matching between cases and controls was not feasible; therefore, we used unconditional logistic regression with adjustment for the matching variables. Several methodological papers have noted that unconditional regression with covariate adjustment can yield valid and unbiased estimates in such scenarios, particularly when matching is not extensive or when some loss of pairs is expected in the analysis phase [35,36]. Moreover, the use of unconditional logistic regression in this study provided greater flexibility to include additional covariates and to retain unmatched observations, thereby preserving sample size and statistical power. However, conditional logistic regression might have been more appropriate for a fully matched design.

Future studies should address these limitations by recruiting larger, population-based samples, incorporating objective exposure assessments—such as air-quality monitoring or biological markers—and employing longitudinal or interventional study designs. These approaches will provide stronger evidence for causal relationships and improve the generalizability of the findings to broader populations.

### Implications and recommendations

The study findings suggest that several lifestyle and environmental factors significantly influence cancer risk among Bangladeshis. These include the use of wood or kerosene as cooking fuel, the consumption of raw or overcooked food, poor oral hygiene, and frequent exposure to mosquito repellents. Addressing these risk factors through targeted public health interventions could play an important role in reducing cancer incidence.

Policy recommendations include promoting the adoption of cleaner household fuels through government-supported energy programs, improving household ventilation systems to reduce indoor air pollution, and raising awareness of safe cooking practices through nutrition education campaigns. Strengthening oral health promotion at the community level could encourage regular dental hygiene and preventive care. Finally, reducing the reliance on chemical mosquito repellents by expanding access to insecticide-treated nets and encouraging environmentally safe alternatives would help limit harmful exposures. Together, these interventions have the potential to mitigate cancer risk and improve population health outcomes in Bangladesh.

To reduce household exposure to indoor air pollution, the government could promote the transition to cleaner cooking fuels, such as liquefied petroleum gas or biogas, particularly in rural areas where biomass use remains high. This may be achieved through targeted subsidy programs, micro-financing options for low-income families, and awareness campaigns delivered via community health platforms. In addition, improvements to household ventilation systems, such as encouraging separate kitchen structures or smoke outlets in housing policy, can further help mitigate the health risks associated with traditional cooking practices.

Public health programs should also incorporate education on safe food preparation and hygiene practices, particularly emphasizing the risks of consuming improperly cooked meat or fish. Nutrition education, delivered through schools, health centers, or community workers, can help raise awareness of these issues in both urban and rural settings. Similarly, integrating oral health promotion into existing primary health care services may improve daily dental hygiene behaviors, especially if accompanied by community-led campaigns and preventive services such as mobile dental check-ups.

To address the risks associated with chemical mosquito repellents, expanding access to insecticide treated bed nets and promoting safer, environmentally friendly alternatives should be prioritized. Public messaging campaigns can help shift behavior away from frequent repellent use, especially in households with children or those at risk of chronic illnesses.

However, the feasibility and cultural acceptability of these interventions should be carefully considered. Barriers such as affordability of cleaner fuels, traditional cooking practices, and preferences for repellent use may limit widespread adoption. Identifying and engaging key stakeholders, including households, community leaders, health professionals, and policymakers, will be essential to ensure that interventions are practical, culturally appropriate, and sustainable.

## Supporting information

S1 Fig Percentage distribution of lifestyle components among cases and controls. (JPEG)

S2 Fig Percentage of case and control with frequent exposure to environmental components. (JPG)

S1 Data De-identified dataset used in this study. (SAV)
