# Supplementary figures and images for: Lifestyle and environmental risk factors associated with cancer: A case-control study in Bangladesh
Source: PLoS One. 2026 Jan 28;21(1):e0328745. doi: 10.1371/journal.pone.0328745 (PMC12851481; doi:10.1371/journal.pone.0328745)

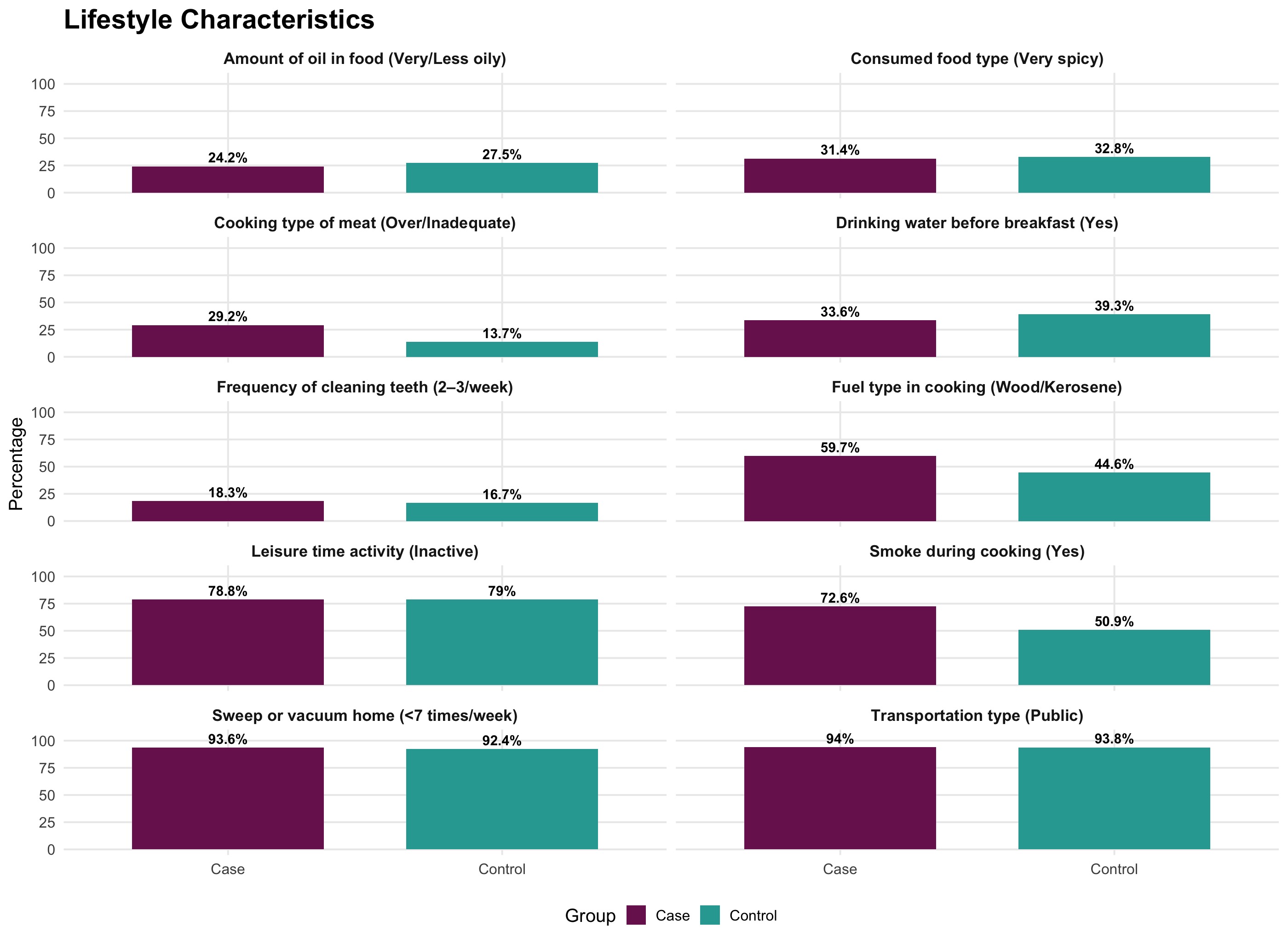

Supplement: S1 Fig — (JPEG) [file pone.0328745.s001.jpeg]

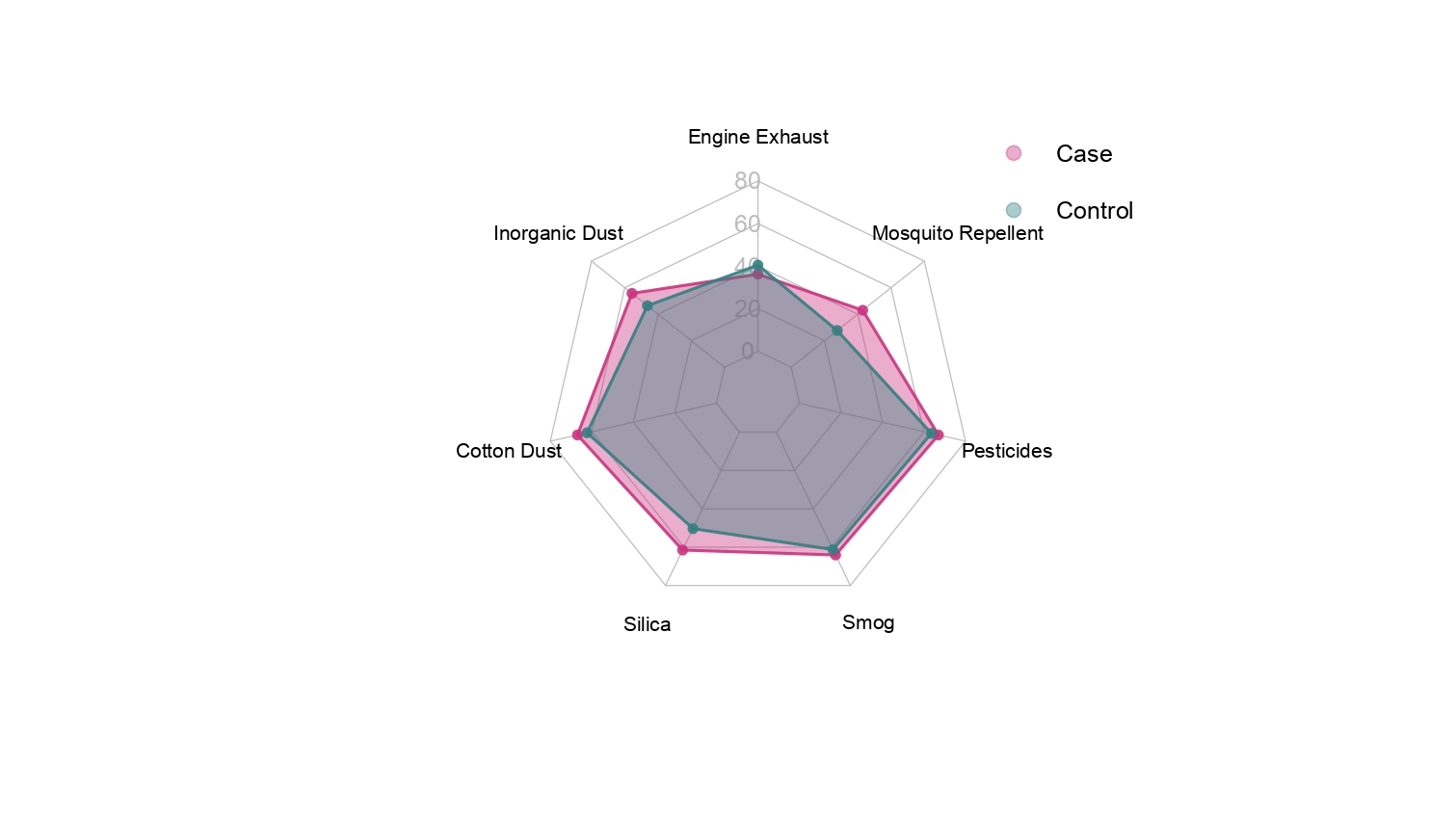

Supplement: S2 Fig — (JPG) [file pone.0328745.s002.jpg]
